# Are Canadian-born Major League Baseball players more likely to bat left-handed? A partial test of the hockey-influence on batting hypothesis

**DOI:** 10.1371/journal.pone.0195252

**Published:** 2018-05-02

**Authors:** John Cairney, Daniele Chirico, Yao-Chuen Li, Emily Bremer, Jeffrey D. Graham

**Affiliations:** 1 Faculty of Kinesiology & Physical Education, University of Toronto, Toronto, Ontario, Canada; 2 Infant and Child Health Lab, Department of Family Medicine, McMaster University, Hamilton, Ontario, Canada; 3 Exercise Physiology and Cardiovascular Health Lab, Division of Prevention and Rehabilitation, University of Ottawa Heart Institute, Ottawa, Ontario, Canada; 4 Child Health Research Center, Institute of Population Health Sciences, National Health Research Institutes, Zhunan, Miaoli County, Taiwan; 5 Department of Kinesiology, McMaster University, Hamilton, Ontario, Canada; University of Denver, UNITED STATES

## Abstract

It has been suggested that Canadian-born Major League Baseball (MLB) players are more likely to bat left-handed, possibly owing to the fact that they learn to play ice hockey before baseball, and that there is no clear hand-preference when shooting with a hockey stick; approximately half of all ice hockey players shoot left. We constructed a database on active (i.e., October, 2016) MLB players from four countries/regions based on place of birth (Canada, United States of America [USA], Dominican Republic and South Asia [i.e., Japan, Taiwan and South Korea]), including information on which hand they use to bat and throw. We also extracted information on all Canadian-born MLB players, dating back to 1917. Our results confirm that the proportion of left-handed batters born in Canada is higher when compared to the other countries selected; also, since 1917, the proportion of Canadian MLB players who bat left has been consistently higher than the league average. We also compared the proportion of left-handed batters in Canada with players born in states in the USA grouped into high, average and low based on hockey participation. The proportion of MLB players born in states with a high level of hockey participation were more likely to bat left, although the differences were significant at trend level only (p < .10). Lastly, we found that while Canadians were more likely to bat left-handed, this did not correspond with a greater left-hand dominance, as determined by throwing hand. In conclusion, the present study confirms that Canadian-born MLB players are more likely to bat left-handed when compared to American, Dominican Republic and South Asian-born MLB players, providing partial support for the hockey influence on batting hypothesis.

## Introduction

Research has demonstrated that batting titles are won more often by left handed MLB hitters in comparison to those who bat right [1], which suggests there is a clear advantage to batting left handed in Major League Baseball (MLB) [2]. Batting averages are found to be higher among left-handed batters as a group as well [2, 3]. Conventional thinking maintains that the advantage is due to the batter being closer to first base, which increases the likelihood of making it there before a throw can be made. The fact that left-handed batters face more right-handed pitchers than right-handed batters face left-handed pitchers has also been identified as one reason for better performance in this group. For example, left-handed batters who are right-hand dominant may be less susceptible to off-speed pitches or breaking balls, as their dominant eye (i.e., right eye, assuming right-eye and right-hand dominance) is closer to the pitcher. While there are many possible explanations, the fact remains, batting left confers a statistical advantage.

Although there is some variability across countries, the prevalence of left-handed dominance in North America and Great Britain is estimated to be between 13% in men and 11% in women [4–5]. Interestingly, approximately 25% of MLB players who were active in the 2016 season batted left [6], suggesting that left-handed batters may be selected into the majors at a higher rate than would be expected when compared against population averages. We hypothesize that Canadian-born MLB players are more likely to bat left-handed and that this may be due to the fact that they grew-up playing hockey before they picked up a baseball bat. In ice hockey, unlike baseball, there does not appear to be a clear dominant hand [5]; about half or more of all hockey players across all levels (i.e., National Hockey League, American Hockey League, and Canadian Hockey League) shoot left [6]. By way of example, consider the 2014 Canadian Men’s Olympic ice hockey team. Of the 22 forwards and defensemen on the roster (goalies, n = 3, were excluded), 12 (54.5%) were listed as left-handed shooters [7]. For a hockey player who shoots left (i.e., the left hand is placed lower on the shaft of the stick), it is a fairly natural transition to place the left hand on top when gripping a bat, which in turn orientates the body to the left side of the plate. It is therefore plausible that children who are exposed to ice hockey early in their development as athletes, a sport where there does not appear to be any clear hand dominance with regard to shooting, may be more likely to bat left if and when they choose to play baseball. Based on the above speculation, we coin this the *hockey influence on batting hypothesis*.

The hockey influence on batting hypothesis is consistent with action-theory, which focuses on the interactions among person, task and environment in shaping sport performance [8]. In this instance, the development of a skill in one sport (i.e., ice hockey), affects the development of a specific skill (i.e., left-hand batting) in another sport (i.e., baseball), through both cultural (e.g., popularity of hockey) and social (e.g., coaching, participation in the sport) influences. Taken for granted in this hypothesis is the interest, motivation and affect of the participant: we assume in this context, playing hockey and baseball is desired and the individual is motivated to practice and play.

Other, anecdotal support for the hockey influence on batting hypothesis comes from media reports on the development of youth baseball in the Czech Republic [9]. The increasing popularity of baseball in that country, and the fact that the national team is now ranked 13^th^ in the world [10], have brought increasing attention to the development of young talents in that country [9]. Coaches from the North America working in that system have commented both on the large number of left-handed batters and on how the swings of many of the youth resemble that of a hockey shot. Like Canada, ice hockey is a very popular sport among youth in the Czech Republic.

While compelling, the hypothesis has yet to be formally tested. Using archival data, we examined four research questions. (1) Are Canadian-born players currently active in the MLB more likely to bat left-handed when compared to players born in the USA and players born outside of North America? By including players born outside the United Sates, we can test for the possibility of a selection effect: foreign players are selected into the MLB because among other skills and attributes, the skill which they can bat left is considered to be an advantage. If there are differences in the proportion of left-handed batters from Canada versus other non-North American countries, then the finding is at least consistent with the hypothesis that the higher number of left-handed batters may be due to having been exposed to ice hockey. (2) Are Canadian-born MLB players more likely to bat left than USA-born MLB players who were born in states where participation in ice hockey is low? While ice hockey is played throughout Canada, it is also popular in some states in the USA, particularly northern (e.g., Wisconsin) and north eastern (e.g., Maine) states. We conducted a partial test of the specificity of the hockey influence on batting hypothesis by taking into account variability in ice hockey participation at the state level where we would expect that if hockey influences hand-dominance in batting, then we would see a higher rate of left-handed batters coming from states where hockey participation is high. (3) Is the higher proportion of left-handed Canadian-born MLB players a recent phenomenon, or has this effect existed throughout the modern history of Canadian participation in the game? While Canadian participation in major league baseball dates back to the 1800s, we decided to examine participation of Canadian-born players from 1917 to present date. The latter date was selected as this marks the formation of the National Hockey League in Canada, and has been identified as a critical date where hockey began to be regarded as the national sport [11, 12]. Lastly, (4) are Canadian left-handed batters more likely to be right-handed throwers? Given that hand dominance is not a factor in shooting in hockey, we would expect that most Canadian-born left-handed batters would throw right.

## Methods

### Data extraction

Players’ country of birth, active or inactive status and hand preference for batting and throwing was determined using two online databases: BaseballReference.com and the official website of Major League Baseball (MLB). All active MLB players whose nationality (i.e., country of birth) was either Canadian, American, Dominican or South-East Asian (i.e., South Korean, Taiwanese and Japanese) at the time of the study (i.e., October, 2016) were included in our database. The latter countries were chosen *a prior* because of the high numbers of players that have played in the MLB from these countries. At present, there are no players from the Czech Republic who play in the majors or from other countries where ice hockey is widely played such as Finland and Germany, reflecting the fact that the rise in interest in baseball is relatively recent. An active MLB player was defined as playing at least one game in the MLB in the 2016 season. Both position players and pitchers were included because the hypothesis is about batting-hand preference, regardless of what position the player currently plays. Also, pitchers in the National League are part of the team batting lineup so to exclude pitchers from the analysis would result in a bias for one half of the League. For American-born players, we also identified the state in which the player was born. All states were then stratified into tertiles based on the participation rates of players registered with USA Hockey, adjusted by each state’s population. Data on the number of registered ice hockey players for the 2015–2016 hockey season in each state were retrieved from the website of USA Hockey [13], and the populations of all states in 2016 were retrieved from the Bureau of the Census, USA [14]. All players with dual-citizenships to the USA were excluded. There were no players from other countries who had dual-Canadian citizenships. When players were identified as “switch” hitters (i.e., they bat from right and left), we categorized them as left-handed batters. However, we also conducted the analysis excluding switch hitters to determine whether the results were affected by this categorization. Finally, we extracted data from the same source for all MLB players whose nationality was Canadian from 1917 to the end of the 2016 regular season so we were able to examine the relative proportion of left-handed batters on an historical and contemporary cohort. Given that all of the data for this analysis is publically available, and that we do not report any individual names of players or report samples sizes less than 5, research ethics approval on this project was not required.

### Statistical analysis

All statistical analyses were conducted using SPSS version 22.0 [15]. In order to test the “*hockey influence on batting hypothesis*”, a series of chi-square tests were used to investigate differences in hand-preference for batting, country of birth and state of birth. Taking into account the relatively small number of cases in some analyses, Cramer’s V was used to estimate effect sizes (ES) [16]. Different criteria for small, medium, and large ES were employed as the interpretation of Cramer’s V is contingent on the degrees of freedom in the analysis [16]. In this study, Cramer’s V values of .07, .21, and .35 indicate small, medium, and large ES, respectively, with df = 2, whereas Cramer’s V values of .06, .17, and .29 indicate small, medium, and large ES, respectively, with df = 3.

## Results

In the first part of the analysis (see Table 1) we examined whether the proportion of left-handed batters born in Canada was higher than players born in the USA, Dominican Republic and Asia (i.e., Japan, South Korea and Taiwan) among professional baseball players active in the MLB as of October 2016. We grouped Japan, South Korea, and Taiwan together owing to the small number of players from each country. The results show that 9 of 13 (69%) active Canadian-born players bat left, which is more than twice as many from Asia (33.3%) and the Dominican Republic (29.9%), and almost 33% higher than players born in the USA (X^2^ = 8.67, df = 3, p < .05; Cramer’s V = .09, p < .05). If we exclude switch hitters the results remain largely unchanged, although the number of Dominican players who bat left declines to 15.3% (n = 17); the proportion of Canadian-born non-switch hitting left-handed batters is 66.7% (n = 8) and the difference between groups remains statically significant (X^2^ = 21.68, df = 3, p < .001; Cramer’s V = .15, p < .001).

**Table 1:** Proportion of Canadian-born versus American-, Dominican Republic- and South-East Asian-born active MLB players who bat left or right.

	N	Bat Left % (n)	Bat Right %(n)
**Canadian-Born**	13	69.2% (9)	30.8% (4)
**American-Born**	975	36.7% (358)	63.3% (617)
**Asian-Born (Japan, South Korea, Taiwan)**	21	33.3% (7)	66.7% (14)
**Dominican Republic-Born**	134	29.9% (40)	70.1% (94)

Note: Switch-hitters are included as left-handed batters

**Fig 1:**
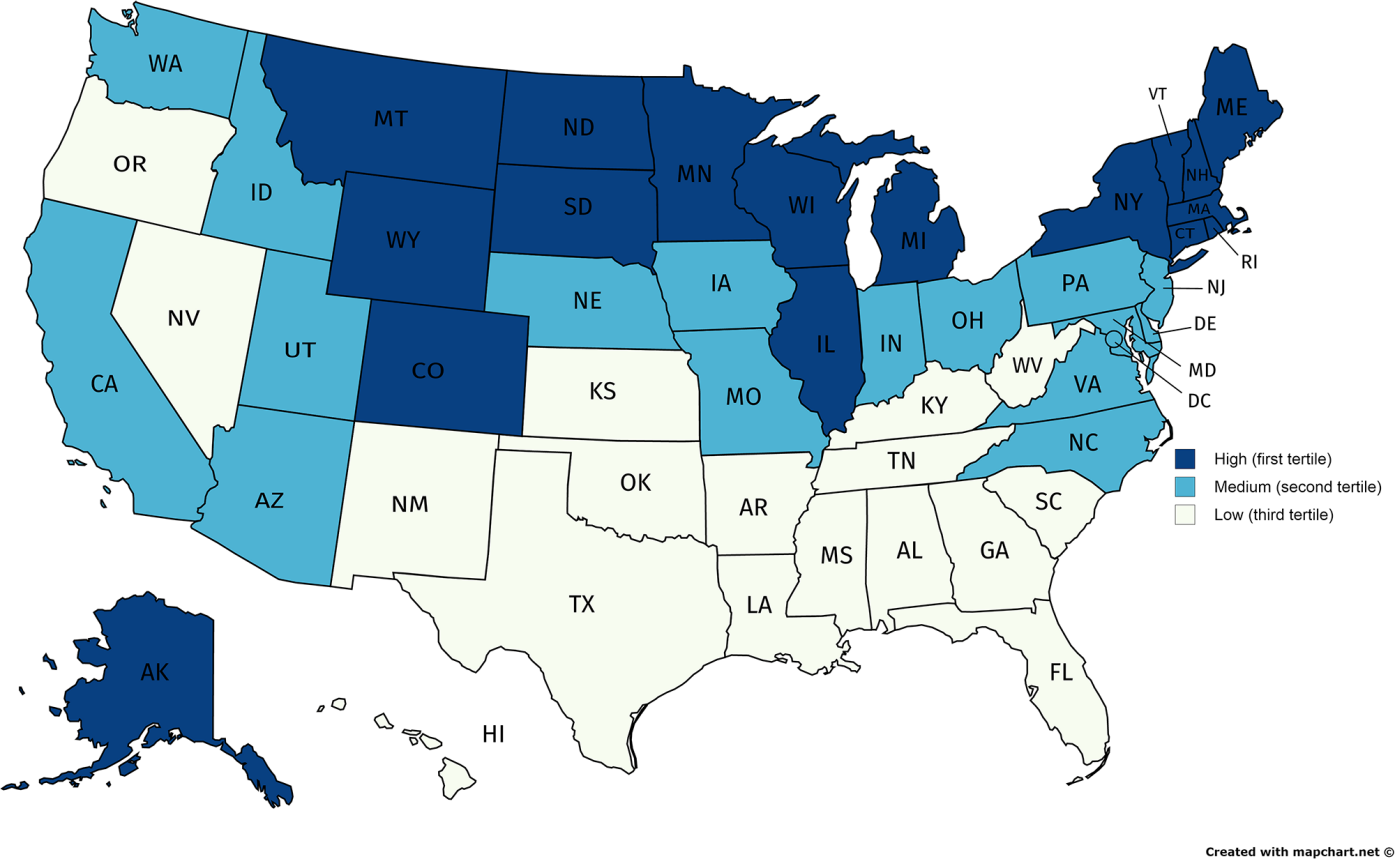
Descriptive statistics of USA hockey participation. States divided into low, medium and high participation levels in USA hockey.

Next, we examined differences in the proportion of left-handed batters between Canadian- and USA-born players, categorizing the latter into categories based on hockey participation rates at the state level in the USA. Using the total participation rates from USA Hockey for each state, we divided all 51 states into high, average, and low participation based on the tertiles for the whole distribution for comparison purposes (see Fig 1). Total participation rates include children, youth and adults from all ages (i.e., 6 and under; 7 to 18, 19 and over) [13]. We then grouped players based on whether they were born in high, average, and low participation states. North eastern and central states that border Canada had the highest rates of participation in ice hockey; overall, hockey participation rates tend to decline moving from north to south.

While there is a trend toward higher proportions of left-handed batters born in states where hockey participation is higher (39.1% compared to 35% in states with the lowest participation rates), the proportion of left-handed batters is lower than Canadian-born players (see Table 2), although the difference is not statistically significant (X^2^ = 6.88, df = 3, p = .08; Cramer’s V = .08, p = .08). Again, excluding batters who switch hit does not affect the results (X^2^ = 6.99, df = 3, p = .07; Cramer’s V = .08, p = .10).

**Table 2:** Proportion of Canadian-born versus American-born active MLB players who bat left or right by region of the United States defined by hockey participation.

	N	Bat Left % (n)	Bat Right % (n)
**Canadian-Born**	13	69.2% (9)	30.8% (4)
**American-Born by Region of the Country Based on Ice Hockey Participation**			
**High**	128	39.1% (50)	60.9% (78)
**Average**	418	37.8% (158)	62.2% (260)
**Low**	429	35.0% (150)	65.0% (279)

Note: Switch-hitters are included as left-handed batters

In the next part of the analysis, we examined the proportion of left-handed batters among all Canadian MLB players who have played in the majors since 1917 (n = 154). The results showed that the proportion of left-handed batters was 53.2% (n = 82) when switch hitters were included as “lefties” and 51.7% (n = 77) when they were excluded.

Finally, the results comparing throwing hand among all left-handed batters are presented in Table 3. Despite no statistical significance found (X^2^ = 5.59, df = 3, p>.05; Cramer’s V = .12, p = .13), a higher proportion of right-handed throwers among left-handed batters was found in Canada (66.7%), Asia (71.4%) and Dominican Republic (62.5%), while a higher proportion of right-handed throwers among left-handed batters in the USA was not evident (52.4%). However, when switch hitters were excluded from analysis, there was a significantly greater proportion of left-handed batters in Canada (62.5%) and Asia (71.4%) who were found to be right-handed throwers, compared to that in the USA (41.2%) and Dominican Republic (17.6%) (X^2^ = 8.03, df = 3, p < .05; Cramer’s V = .15, p < .05). We further analyzed the difference in the proportion of left-handed batters throwing right between the states with different hockey participation (also shown in Table 3). There was no significant difference in the proportion between the states with high, medium, and low hockey participation (X^2^ = .48, df = 2, p>.05; Cramer’s V = .04, p = .79), indicating the percentage of players throwing right was consistently lower among all lefty batters across three regions. When switch hitters were excluded from analysis, the result remained the same, although the proportion of left-handed throwers increased 5% to 7% in all three regions (Results not shown here).

**Table 3:** Number and proportion of left- and right-handed throwers among left-handed batters.

	Left-Handed Batters (Note: Switch Hitters included)	
	Left-Hand Throwing	Right-Hand Throwing
**Canada-Born (n = 9)**	3 (33.3%)	6 (66.7%)
**American-Born (n = 357)**^a^	187 (52.4%)	170 (47.6%)
**American-Born by Region of the Country Based on Ice Hockey Participation**		
**High (n = 50)**	27 (54.0%)	23 (46.0%)
**Average (n = 158)**^a^	79 (50.3%)	78 (49.7%)
**Low (n = 150)**	81 (54.0%)	69 (46.0%)
**Asian-Born (n = 7)**	2 (28.6%)	5 (71.4%)
**Dominican Republic-Born (n = 40)**	15 (37.5%)	25 (62.5%)

^a^ One player who can throw using both hands was excluded

## Discussion

Overall, our analysis of archival data confirms that there is a higher proportion of left-handed batters born in Canada that are currently active in the MLB, as compared to American-born players and players born in the Dominican Republic and South-East Asia. Furthermore, this does not appear to be a recent phenomenon; between half and 53% of all Canadian-born MLB players since 1917 have batted left. Based on the current data of this study we cannot ascertain whether this was actually due to playing ice hockey prior to playing baseball; however, it is interesting to note that the proportion of left-handed batters appears to be higher not only in Canada, but also in US states where hockey participation is higher (differences approached significance with a small to medium ES [*p* = .08, Cramer’s V = .08, p = .08]). There are simply not enough MLB players born in countries where ice hockey is also popular (e.g., Scandinavia, Russia) to further test this hypothesis. It is also interesting to note that with the exception of players born in the USA, most left-handed batters throw right, suggesting that left-hand dominance is not a major factor in determining batting preference. In support of this finding, a recent study demonstrated that in bilateral manual sport-related tasks, meaning tasks that require use of both hands (e.g., batting, shooting with a hockey stick), there appears to be a higher percentage of left lateral preference (i.e., batting left) compared to unilateral manual tasks such as throwing darts, or holding a racquet [15]. Furthermore, the correlation between handedness and lateral preference in tasks that require bimanual control appears to be quite weak, particularly for tasks such as swinging a bat and holding a hockey stick [15]. Interestingly, in the present study, between 63 and 67% of Canadian-born players throw right and bat left, depending on how switch hitters are classified. Collectively, these findings suggest that factors other than hand dominance influences left lateral preference in Canadian MLB players. This is at least consistent with the fact that hockey may play an influential role in determining lateral preference in Canadian baseball players.

In addition to strategic advantages of left-handed batting (e.g., being closer to the first base), there is a plausible explanation from a functional perspective for the hockey influence on batting hypothesis. It was reported that over the last 10 years, the average sale of hockey sticks by Bauer Hockey in Canada was 65% left-handed compared to 35% right-handed sticks [17]. As Canadian baseball players may also have more experience playing ice hockey when they were young, or even prior to playing baseball, their bodies may develop a physically “adaptive response” to shoot left. In hockey, a left-handed shooter places their left hand on the lower shaft of the stick and right hand at the top, and the blade of the stick is touching the ice near their left foot. Therefore, when transitioning to a baseball stance, a natural progression may be a left-handed batting preference whereby the right is at the bottom and the left is on top. As a result, rotation along the vertical axis (i.e., right) remains consistent. Nevertheless, due to a lack of direct evidence (e.g., the comparison of the prevalence of left- and right-handed batters between left- and right-handed hockey players), further research is needed to test the hypothesis of “adaptive response”, which will further support the hockey influence on batting hypothesis. Furthermore, there appears to be no clear hand preference in hockey, with a majority of players likely basing the decision on comfort or perhaps modeling after their favourite player. However, it is suggested that proper technique for generating power for a slap shot or wrist shot, and better control and accuracy originates from the top hand; right hand dominant hockey players may feel more comfortable holding the top of their stick with their right hand as it provides more control of the stick, particularly when required to hold the stick with one hand while engaging opponents [18].

Motor learning, however, is only part of the explanation. Based on action-theory [10], one has to also consider the socio-cultural context along with individual factors, such as motivation and affect, in which these sports are played. Ice hockey is widely considered to be Canada’s national sport and a symbol of cultural identity (see pg. 209 in [19]), and large numbers of children and youth play not only the ice version of the game, but also land-based versions which is sometimes called ball or “street” hockey as a rubber ball or tennis ball is substituted for a puck and the game is played on a flat, non-slippery surface such as a gym floor, or on a paved road. At the same time, baseball is also extremely popular in Canada and historians have argued that prior to the 1920s and the institution of indoor ice arenas, baseball rivaled ice hockey and other sports as Canada’s national pastime [11]. Many children and youth therefore, play both sports (e.g., alternating based on the season—ice hockey in the winter, baseball in the summer).

With increasing pressure to specialize in a single sport, which is common throughout North America and Europe [20], the obsession with ice hockey as a national game, and the desire of some parents to ensure their children play at high (i.e., varsity/collegiate), even professional levels, children are often put into hockey at a very early age (e.g., under 6) [20]. This early exposure to either ice or ball/street hockey may influence participation and skill development in other sports. In 2005 for example, more than twice as many children aged 5 to 15 played hockey compared with baseball (11% compared to 5% [21]. As noted previously, because there is no clear hand-dominance in shooting, coaches do not correct hand-placement when holding a stick. The progression therefore from shooting in hockey to batting in baseball (i.e., adaptive response) unfolds as described. In this sense, a motor learning effect interacts with cultural (i.e., dominance of hockey as a national sport) and social (i.e., trends to early specialization and coaching practices) factors, leading to a specific skill advantage when one crosses over from one sport to another. This is consistent with action theory, which postulates complex interactions between motor skills, individual and social/environmental contextual influences. Implicit in all this is that the individual has a desire and interest to play both sports and in the case of professional baseball, the psychological and emotional make-up, along with skill, to perform at a high level of competition.

We would be re-miss however, if we did not point out that this confluence of individual and environmental effects affecting the development of left-handed batting in Canadians is not static. Hockey participation rates in Canada have been on the decline for over a decade owing to a number of factors including the high cost of equipment, parental concerns over head injury risk, and the changing ethnic composition of the country through immigration (i.e., families immigrating to Canada from countries where ice hockey is not played) [22]. In this sense, in addition to social and cultural contextual influences, historical period is also a determining factor.

Although our findings are consistent with the hockey influence hypothesis, there are some findings that require further investigation. First, our finding that MLB players who were born in US states where hockey participation rates are above average is intriguing. While this would be consistent with the hypothesis, the differences in prevalence observed were not statistically significant. This may be a power issue as there is an unequal distribution of sample sizes in three regions and a smaller sample size in the region with a high rate of hockey participation. Also, the findings that 71% of right-handed throwers born in Asia (i.e., Japan, Taiwan and South Korea) bat-left is also an intriguing finding and one that forces a consideration of other sports or activities that may promote this mixed-laterality of batting and throwing. We selected these countries a priori because the chance of exposure to ice hockey is very low. If not hockey, then why do we see a similar pattern of mixed-laterality to Canadian-born MLB players? One explanation may be that coaches in this country promote the practice of batting left at early developmental stages of the sport [23]. These countries also have large populations and large numbers of children and youth playing baseball. The statistical probability of finding a left-handed batter in the developmental system is high and these players are selected into the MLB at a higher rate given their unique skill set. Further research is required to investigate this phenomenon.

Our findings are at best only a partial test of the hockey influence on batting hypothesis. There are several limitations in our data that should be addressed in future research. First, place of birth is not the most sensitive indicator to determine exposure to hockey. We would need to know if indeed players actually played hockey as children and youth, and whether they played or learned to shoot in ice or road hockey prior to learning to bat in baseball. Also, place of birth does not take into account the fact that where a person was born may not be the same place as where they grew up. We lacked the data to be able to determine if the player lived in a place where ice hockey was popular during their childhood and adolescence, periods when exposure to both sports would have occurred. With these considerations in mind, further research should involve direct surveying of Canadian baseball players who play at the professional level where direct questions regarding place of birth, experiences with hockey growing up, and which sport they played first can be measured. Finally, at least among active MLB players, the numbers of players born in Canada is relatively small, as are numbers of players born in countries such as Japan and Taiwan. Future work may expand the sample to include minor league and NCAA-level athletes, or other bi-manual sports (e.g., golf or lacrosse) to see if the trends observed here hold. Examination of other ball and bat games, for example cricket, could be examined as well. In the latter case, it would be interesting to see if exposure to sports such as field hockey, influences handedness in batting.

Notwithstanding these limitations, this is the first empirical test we can find of the hockey influence on batting hypothesis. Our results are consistent with the explanation and warrants further study of the phenomena.

## Supporting information

S1 Appendix Exemption from ethics review. Letter of confirmation of exemption from ethics review from the University of Toronto.
(PDF)
