# Supplementary material for: Are Canadian-born Major League Baseball players more likely to bat left-handed? A partial test of the hockey-influence on batting hypothesis
Source: PLoS One. 2018 May 2;13(5):e0195252. doi: 10.1371/journal.pone.0195252 (PMC5931458; doi:10.1371/journal.pone.0195252)
Supplement: S1 Appendix — Letter of confirmation of exemption from ethics review from the University of Toronto. (PDF) [file pone.0195252.s001.pdf]

UNIVERSITY OF TORONTO

OFFICE OF THE VICE-PRESIDENT, RESEARCH AND INNOVATION

December 8, 2017

John Cairney, PhD
Faculty of Kinesiology & Physical Education
University of Toronto

Dear Dr. Cairney,

Re: Your project entitled “Are Canadian-born Major League Baseball Players More likely to Bat Left-handed? A partial test of the hockey-influence on batting hypothesis”

I am writing to advise you that based on the description of your project with the above title, I have determined that it is exempt from ethics review at our office.

This is based on my understanding that the study is solely based on publicly available data. Our guidelines, TCPS, allows exemption from ethics review, research based exclusively on publicly available data.

Please find the relevant article in the **Tri-Council Policy Statement,** Article 2.2:

http://www.pre.ethics.gc.ca/eng/policy-politique/initiatives/tcps2-eptc2/chapter2-chapitre2/

We wish you the best of luck with your undertaking.

Sincerely,

Daniel Gyewu, M.Sc.
Research Ethics Board Manager- Health Sciences

OFFICE OF RESEARCH ETHICS
McMurrich Building, 12 Queen's Park Crescent West, 2nd. Floor, Toronto, ON M5S 1S8 Canada
Tel: +1 416 946-3273 • Fax: +1 416 946-5763 • ethics.review@utoronto.ca • http://www.research.utoronto.ca/for-researchers-administrators/ethics/
